# IGF-1-Overexpressing Mesenchymal Stem/Stromal Cells Promote Immunomodulatory and Proregenerative Effects in Chronic Experimental Chagas Disease

**DOI:** 10.1155/2018/9108681

**Published:** 2018-07-24

**Authors:** Daniela N. Silva, Bruno S. F. Souza, Carine M. Azevedo, Juliana F. Vasconcelos, Paloma G. de Jesus, Malena S. Feitoza, Cassio S. Meira, Gisele B. Carvalho, Bruno Raphael Cavalcante, Ricardo Ribeiro-dos-Santos, Milena B. P. Soares

**Affiliations:** ^1^Center for Biotechnology and Cell Therapy, São Rafael Hospital, Salvador, BA, Brazil; ^2^Gonçalo Moniz Institute, FIOCRUZ, Salvador, BA, Brazil; ^3^National Institute of Science and Technology for Regenerative Medicine, Rio de Janeiro, RJ, Brazil

## Abstract

Mesenchymal stem/stromal cells (MSCs) have been investigated for the treatment of diseases that affect the cardiovascular system, including Chagas disease. MSCs are able to promote their beneficial actions through the secretion of proregenerative and immunomodulatory factors, including insulin-like growth factor-1 (IGF-1), which has proregenerative actions in the heart and skeletal muscle. Here, we evaluated the therapeutic potential of IGF-1-overexpressing MSCs (MSC_IGF-1) in a mouse model of chronic Chagas disease. C57BL/6 mice were infected with Colombian strain *Trypanosoma cruzi* and treated with MSCs, MSC_IGF-1, or vehicle (saline) six months after infection. RT-qPCR analysis confirmed the presence of transplanted cells in both the heart and skeletal muscle tissues. Transplantation of either MSCs or MSC_IGF-1 reduced the number of inflammatory cells in the heart when compared to saline controls. Moreover, treatment with MSCs or MSC_IGF-1 significantly reduced TNF-*α*, but only MSC treatment reduced IFN-*γ* production compared to the saline group. Skeletal muscle sections of both MSC- and MSC_IGF-1-treated mice showed a reduction in fibrosis compared to saline controls. Importantly, the myofiber area was reduced in *T. cruzi*-infected mice, and this was recovered after treatment with MSC_IGF-1. Gene expression analysis in the skeletal muscle showed a higher expression of pro- and anti-inflammatory molecules in MSC_IGF-1-treated mice compared to MSCs alone, which significantly reduced the expression of TNF-*α* and IL-1*β*. In conclusion, our results indicate the therapeutic potential of MSC_IGF-1, with combined immunomodulatory and proregenerative actions to the cardiac and skeletal muscles.

## 1. Introduction

In the field of regenerative medicine, mesenchymal stem/stromal cells (MSCs) are promising tools for the development of novel advanced therapy medicinal products to treat chronic inflammatory diseases [1]. MSCs can be easily obtained from different sources, including the bone marrow, adipose tissue, and umbilical cord tissue, allowing for the development of both autologous and allogeneic therapies [2]. Several clinical trials that have been performed have demonstrated the safety of the clinical application of MSCs [3]. In order to improve the therapeutic effects of MSCs, genetic modification for the overexpression of specific growth factors is currently being investigated [4, 5].

The release of trophic paracrine factors by MSCs has been associated with many of the proregenerative and immunomodulatory effects observed in the context of MSC-based therapies [2]. One of such growth factors is insulin-like growth factor-1 (IGF-1), which has been shown to exert proregenerative actions in skeletal muscle, promoting muscle cell proliferation and differentiation, histological recovery of muscle fiber type and size, and functional improvements [6, 7]. Moreover, IGF-1 induces myocyte hypertrophy and satellite cell activation and increases protein synthesis in differentiated myofibers [8]. In heart tissue, IGF-1 was shown to improve engraftment of MSCs, promoting neovascularization and inhibiting cardiomyocyte death [9, 10].

Chagas disease is an infectious disease caused by the intracellular parasite *Trypanosoma cruzi*. Previously confined to the Latin American region, Chagas disease has now spread to other continents due to population migration [11]. Cardiac complications occur in approximately 30% of infected subjects, which may present arrhythmias and heart failure [12]. Tissue analysis reveals the presence of chronic myocarditis, myocytolysis, and an intense interstitial fibrosis, as the result of a combination of persistent parasitism, microvascular inflammation, neurogenic dysfunction, and autoimmune responses [13]. These processes are also observed in the mouse model of Chagas disease caused by a myotropic *Trypanosoma cruzi* strain, which develops a progressive inflammatory response and fibrosis in the heart, along with an intense skeletal myositis, in the chronic phase of infection [14]. Modulation of the exacerbated inflammatory response in combination with the stimulation of endogenous regeneration represents a promising therapeutic approach for Chagas disease.

The ability of MSCs to modulate immune responses and fibrosis has been demonstrated in the context of *T. cruzi* infection in mice [15–18]. We have previously generated and characterized a bone marrow-derived MSC cell line overexpressing IGF-1 [19]. In the present study, we investigated the therapeutic potential of IGF-1-overexpressing MSCs in the experimental model of chronic Chagas disease, by evaluating their immunomodulatory and proregenerative effects in the heart and skeletal muscles.

## 2. Materials and Methods

### 2.1. Animals

Six- to eight-week-old female C57BL/6 mice were used for *T. cruzi* infection. Age-matched naïve mice were kept under the same conditions during the experiments, to serve as uninfected controls. All animals were raised and maintained in the animal facility of the Center for Biotechnology and Cell Therapy, São Rafael Hospital (Salvador, Brazil), and provided with rodent diet and water ad libitum. Animals were handled according to the National Institutes of Health guidelines for animal experimentation. All procedures described had prior approval from the local animal ethics committee under number 012/09 (São Rafael Hospital, Bahia, Brazil).

### 2.2. Culture of IGF-1-Overexpressing MSCs

A genetically modified MSC line with stable overexpression of human IGF-1 (MSC_IGF-1) was previously generated using bone marrow MSCs obtained from GFP transgenic mice [19]. Briefly, MSCs were transduced with a lentiviral vector for overexpression of hIGF-1, and clones were characterized by polymerase chain reaction (PCR) and enzyme-linked immunosorbent assay (ELISA) to assess transgene expression. The cells were also shown to maintain MSCs' characteristics, including phenotypic markers, trilineage differentiation potential, and reduced inhibition of lymphocyte proliferation. MSC_IGF-1 was cultured in Dulbecco's Modified Eagle's Medium (DMEM) supplemented with 10% fetal bovine serum (FBS) and 1% penicillin/streptomycin (all from Thermo Fisher Scientific, Waltham, MA, USA) in a humidity-controlled incubator at 37°C and 5% CO_2_, with complete medium replacement every three days.

### 2.3. *T. cruzi* Infection and Cell Therapy

**Figure 1:**
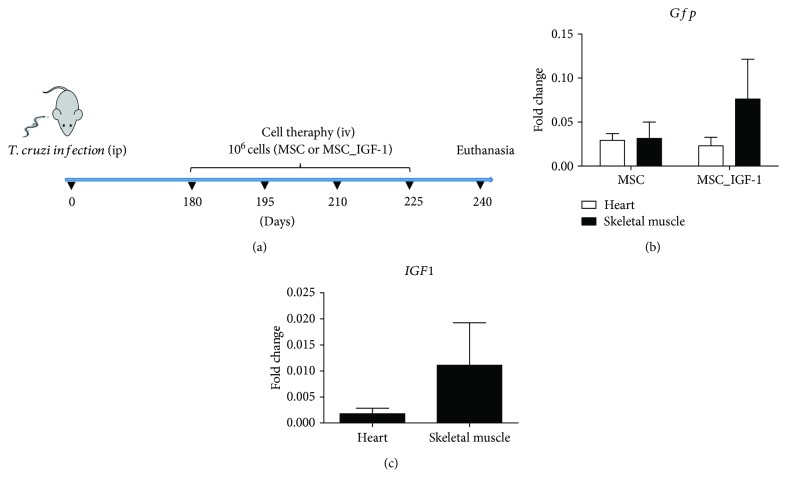
Experimental design and cell tracking. C57BL/6 mice were infected with 1000 *T. cruzi* trypomastigotes (Colombian strain) and treated, during the chronic phase, with 10^6^ MSCs or MSC_IGF-1, intravenously, every 15 days, during 60 days (a). Saline-treated and naïve mice were used as controls. Heart and skeletal muscle tissues were collected 15 days after the last administration of cells for RT-qPCR analysis of GFP (b) or human IGF-1 gene expression (c). Values represent the mean ± SEM of 5–7 animals/group.

Mice were infected by intraperitoneal injection with 1000 trypomastigotes of Colombian *T. cruzi* strain, obtained from culture supernatants of infected LLC-MK2 cells. Six months after the infection, mice were randomly assigned into three groups: MSCs (*n* = 10), MSC_IGF-1 (*n* = 10), or saline (*n* = 8). Age-matched naïve mice were used as normal controls (*n* = 10). Cell transplantation was performed by four intravenous injections of cell suspensions containing either 10^6^ MSCs or MSC_IGF-1, in saline, with an interval of 15 days between each injection. An equal volume of vehicle (100 *μ*L) was used in the saline group (Figure 1(a)).

### 2.4. Morphometric Analyses

Mice were euthanized two months after the initiation of the cell therapy protocol, under anesthesia with ketamine and xylazine. Heart and skeletal muscles were removed and fixed in 10% buffered formalin. Tissue sections were analyzed by light microscopy after paraffin embedding, followed by standard hematoxylin and eosin (H&E) staining. Inflammatory cells were counted using the software Image-Pro Plus v.7.0 (Media Cybernetics, Rockville, MD, USA). The number of inflammatory cells was determined by counting 10 fields (400x magnification) per heart or skeletal muscle section. Sirius Red-stained sections were entirely digitalized using a confocal microscope A1+ (Nikon, Tokyo, Japan). The percentage of fibrosis was determined by analysis of whole sections of heart or skeletal muscle, stained with Sirius Red with a semiautomatic morphometric protocol, using Image-Pro Plus v.7.0 (Media Cybernetics, Rockville, Maryland, USA). Two blinded investigators performed the analyses.

### 2.5. Immunofluorescence Analyses

Skeletal muscle sections of 10 *μ*m were fixed with 4% paraformaldehyde and incubated overnight at 4°C with skeletal myosin primary antibody diluted 1 : 50 (Sigma-Aldrich, St. Louis, MO, USA). On the following day, the sections were incubated for 1 h with secondary antibody anti-rabbit IgG Alexa Fluor 488 conjugate at dilution of 1 : 600 (Thermo Fisher Scientific). Nuclei were stained with 4,6-diamidino-2-phenylindole (VECTASHIELD mounting medium with DAPI H-1200; Vector Laboratories, Burlingame, CA, USA). The presence of fluorescent fibers was determined by observation in the A1+ confocal microscope (Nikon). Quantifications of stained areas were performed in large image captured under 100x magnification, using the Image-Pro Plus v.7.0 software (Media Cybernetics).

### 2.6. Cytokine Measurement

Cytokine concentrations were evaluated in the serum by ELISA, using DuoSet kits for tumor necrosis factor alpha (TNF-*α*), interferon gamma (IFN-*γ*), interleukin 10 (IL-10), and transforming growth factor beta (TGF-*β*), according to the manufacturer's instructions (R&D Systems, Minneapolis, MN, USA).

### 2.7. Real-Time Reverse Transcription Polymerase Chain Reaction (RT-qPCR)

Total RNA was isolated from heart and skeletal muscle samples with a TRIzol reagent (Thermo Fisher Scientific), and concentration was determined by photometric measurement. High-Capacity cDNA Reverse Transcription Kit (Thermo Fisher Scientific) was used to synthesize cDNA of 1 *μ*g RNA by following the manufacturer's recommendations. RT-qPCR assays were performed to detect the expression levels of *Tnf* (Mm00443258_m1), *Ifng* (Mm00801778_m1), *Nos2* (Mm01309898m1), *Arg1* (Mm00475988_m1), *Il1b* (Mm00434228_m1), *Il10* (Mm00439616_m1), *Tgfb1* (Mm00441724_m1), *Ptpcr* (mm01293577_m1), and *Cox2* (Mm01307329_m1). For the detection of GFP and human IGF-1 mRNA, the following primer sequences were used in real-time PCR assays: GFP: 5′-AGCAGAACACCCCCATCG-3′ and 3′-TCCAGCAGGACCATGTGATC-5′ and hIGF-1 5′CCAAGACCCAGAAGGAAGTACA-3′ and 3′-TGGCATGTCACTCTTCACTCC-5′. The RT-qPCR amplification mixtures contained 20 *μ*g template cDNA, TaqMan Master Mix (10 *μ*L), and probes in a final volume of 20 *μ*L (all from Thermo Fisher Scientific). All reactions were run in duplicate on an ABI 7500 Sequence Detection System (Thermo Fisher Scientific) under standard thermal cycling conditions. The mean cycle threshold (Ct) values from duplicate measurements were used to calculate expression of the target gene, with normalization to an internal control, *Gapdh*, using the 2 − DCt formula. Experiments with coefficients of variation greater than 5% were excluded. A nontemplate control (NTC) and nonreverse transcription controls (No-RT) were also included.

### 2.8. Statistical Analyses

Statistical comparisons between groups were performed by Student's *t*-test when comparing two groups and ANOVA followed by a Newman-Keuls post hoc test for multiple comparisons, using a GraphPad Prism program (Software Inc., San Diego, CA, USA) version 5.0. Results were considered significant when *P* < 0.05.

## 3. Results

### 3.1. MSCs and MSC_IGF-1 Are Detected in the Heart and Skeletal Muscles following Transplantation to Chronic Chagasic Mice

A treatment regimen composed of repeated intravenous injections of MSCs or MSC_IGF-1 (Figure 1(a)) was associated with increased mortality (40% for the MSC group and 30% for the MSC_IGF-1 group) in mice chronically infected with *T. cruzi*, due to pulmonary embolism. Surviving mice (*n* = 6 for the MSC group and *n* = 7 for the MSC_IGF-1 group) were euthanized two months following the initiation of the treatment regimen for histological and gene expression analyses and quantification of cytokines (Figure 1(a)).

First, we evaluated the presence of transplanted cells in the heart and skeletal muscles by detection of the transgene mRNAs by RT-qPCR. GFP was used to detect both MSCs and MSC_IGF-1, while hIGF-1 was used to detect MSC_IGF-1. We detected GFP mRNA in the hearts of six out of seven mice from the MSC_IGF-1 group and five out of six mice in the MSC group (Figure 1(b)). While no expression of hIGF-1 was found in mouse hearts from the MSC group, four out of seven mice in the MSC_IGF-1 group were positive for the expression of this mRNA (Figure 1(c)). In the skeletal muscle, all mice treated with MSCs were positive for GFP, while 4 out of 7 mice presented both expressions of GFP and hIGF-1 in mice treated with MSC_IGF-1 (Figures 1(b) and 1(c)).

### 3.2. Cell Therapy with MSCs and MSC_IGF-1 Modulates Cardiac Inflammation and Fibrosis

**Figure 2:**
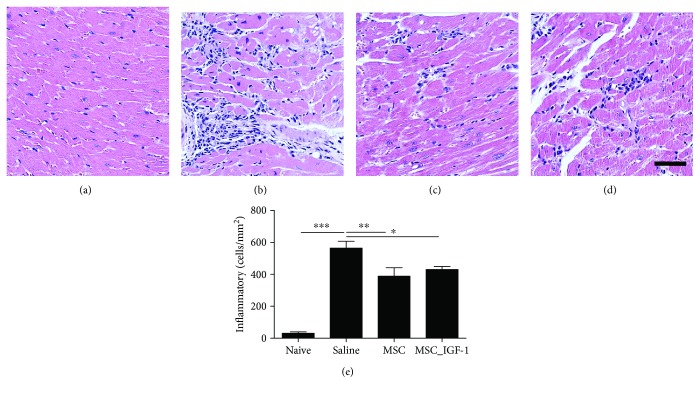
Quantification of inflammatory cells infiltrating the heart. Representative images of heart sections from mice euthanized two months after cell therapy with MSCs, MSC_IGF-1, or untreated controls. Heart sections were stained with H&E, and the number of inflammatory cells was quantified, comparing naïve (a) and infected mice treated with saline (b) MSCs (c) or MSC_IGF-1 (d). Bars = 50 *μ*m. (e) Number of inflammatory cells per mm^2^ in H&E-stained sections. Data represent the mean ± SEM of 5–7 animals/group. ^∗^*P* < 0.05, ^∗∗^*P* < 0.01, and ^∗∗∗^*P* < 0.001.

**Figure 3:**
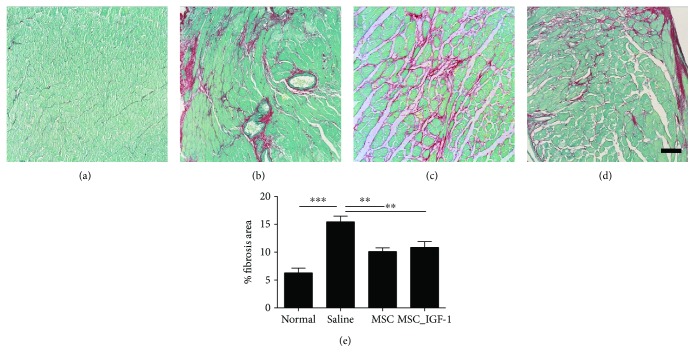
Quantification of cardiac fibrosis. Representative images of heart sections stained with Sirius Red obtained from naive (a) or infected mice treated with saline (b), MSCs (c), or MSC_IGF-1 (d). Bars = 50 *μ*m. (e) Quantification of the percentage of cardiac fibrosis area. Results are expressed as mean ± SEM of 5–7 animals/group. ^∗∗^*P* < 0.01 and ^∗∗∗^*P* < 0.001.

Next, we evaluated the effects of cell therapy by tissue analysis in the hearts of chagasic mice. All mice chronically infected with *T. cruzi* presented intense inflammatory infiltrates in the myocardium, which were mainly composed of mononuclear cells (Figures 2(a)–2(d)). Infiltrating inflammatory cells were quantified, and a significant reduction in the number of cells was measured in both MSC- and MSC_IGF-1-treated mice, when compared to saline-treated controls (Figure 2(e)). Diffuse areas of cardiac fibrosis, distributed along the atria, atrioventricular junction, and ventriculi, were observed in all *T. cruzi*-infected mice and not in all naïve controls (Figures 3(a)–3(d)). A reduction in the fibrotic area was also observed in the hearts of mice treated with either MSCs or MSC_IGF-1, when compared to saline-treated controls (Figure 3(e)).

**Figure 4:**
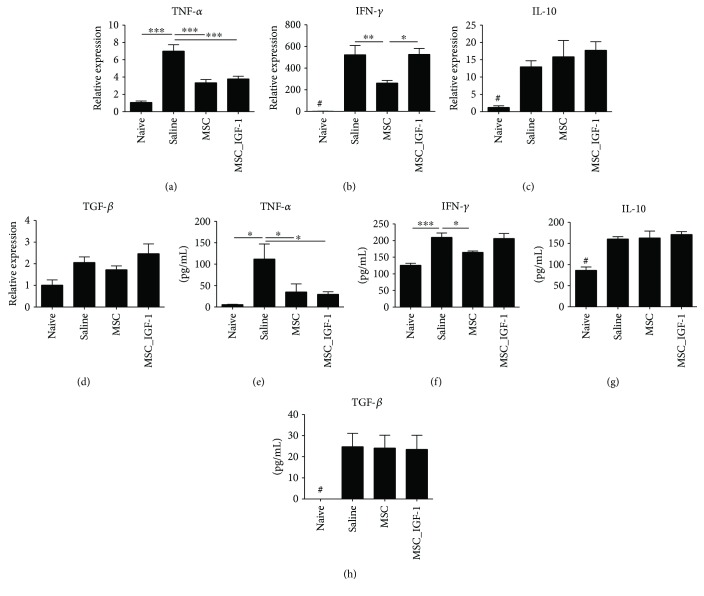
Cytokine evaluation in the heart and serum after cell therapy. Samples or infected mice treated with saline, MSCs, or MSC_IGF-1 were collected two months following the initiation of the cell therapy protocol and analyzed by RT-qPCR in the heart tissue for gene expression (a–d) and by ELISA in the serum for protein quantification (e–h) of TNF-*α* (a, e), IFN-*γ* (b, f), IL-10 (c, g), and TGF-*β* (d, h). Data represent the mean ± SEM of 5–7 mice per group. ^∗^*P* < 0.05, ^∗∗^*P* < 0.01, ^∗∗∗^*P* < 0.001, and ^#^*P* < 0.0001 compared to other groups.

Additionally, the expression levels of proinflammatory and anti-inflammatory genes were evaluated in heart samples. Treatment with MSCs or MSC_IGF-1 produced a statistically significant reduction in the expression of TNF-*α*, while IFN-*γ* gene expression was reduced only in MSC-treated mouse hearts (Figures 4(a) and 4(b)). No significant differences were observed in TGF-*β* or IL-10 gene expression between MSC-, MSC_IGF-1-, and saline-treated groups (Figures 4(c) and 4(d)).

We also investigated the systemic immunomodulatory effects of cell therapy by quantification of cytokines in the serum. Similar to the findings in the heart tissue, the levels of TNF-*α* were reduced significantly in the groups treated with either MSCs or MSC_IGF-1, although IFN-*γ* levels decreased only in the MSC-treated group (Figures 4(e) and 4(f)). No statistically significant differences were observed in TGF-*β* or IL-10 serum levels, when comparing treated mice to saline controls (Figures 4(g) and 4(h)).

### 3.3. Proregenerative and Immunomodulatory Actions of MSC_IGF-1 in the Skeletal Muscle of Mice Chronically Infected with *T. cruzi*

**Figure 5:**
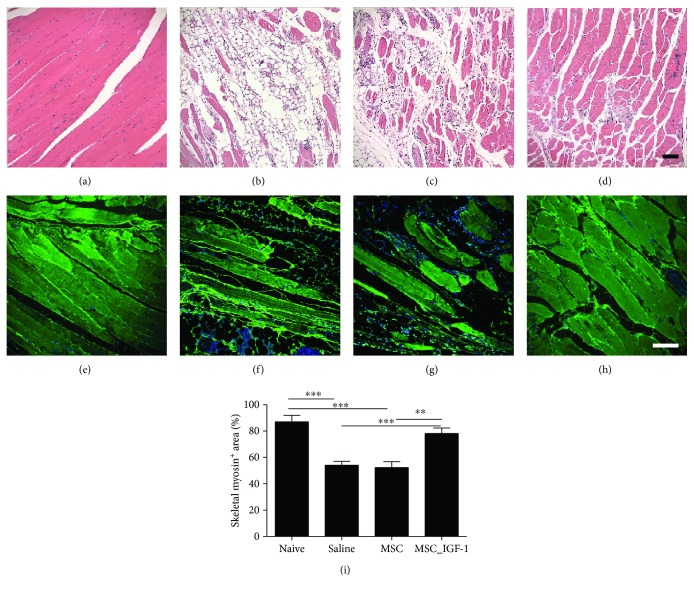
Proregenerative effects of therapy with MSC_IGF-1 in the skeletal muscle. Representative images of skeletal muscle sections stained with H&E, obtained from naïve (a) or infected mice treated with saline (b), MSCs (c), or MSC_IGF-1 (d), showing destruction of myofibers and substitution for fibrosis and adipose tissue in infected mice, when compared to naïve mice, and recovery in MSC_IGF-1-treated mice (d). Skeletal myosin staining in skeletal muscle sections from naïve (e) or infected mice (f) treated with saline (g), MSCs (h), or IGF-1 (i), confirming the enhanced presence of myosin^+^ myofibers in MSC_IGF-1-treated mice. Bars = 100 *μ*m. (j) Quantification of the skeletal myosin^+^ area. ^∗∗^*P* < 0.01 and ^∗∗∗^*P* < 0.001.

Skeletal muscle from naïve mice presented a normal microscopic structure, with preserved myocytes, as observed by H&E staining (Figure 5(a)). In contrast, sections from *T. cruzi*-infected mice of saline and MSC groups presented clear signs of skeletal muscle destruction and substitution for fibrosis and adipose tissue (Figures 5(b) and 5(c)). In MSC_IGF-1-treated mice, however, we observed a marked preservation of the myofibers (Figure 5(d)). This finding was confirmed by quantification of the area occupied by myofibers, visualized by positive staining for skeletal myosin (Figures 5(e)–5(h)). Naïve mice had a significantly higher myosin^+^ area than saline- or MSC-treated mice and presented a similar pattern to MSC_IGF-1 mice (Figure 5(i)).

**Figure 6:**
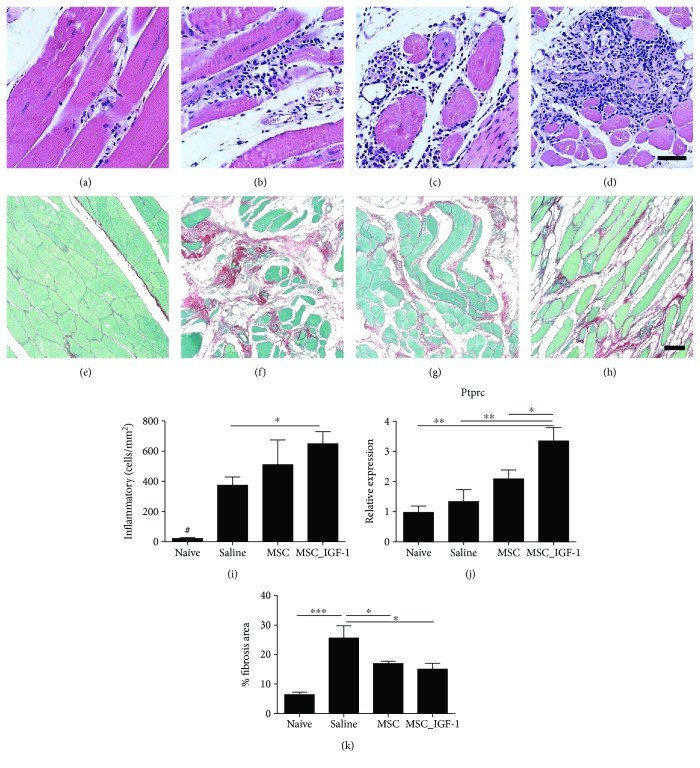
Quantification of inflammatory infiltrates and fibrosis area in the skeletal muscle. Representative images of skeletal muscle sections stained by conventional H&E stain for analysis of inflammatory infiltrates in naïve (a) or infected mice submitted to treatment with saline (b), MSCs (c), or MSC_IGF-1 (d). Representative images of Sirius Red staining for quantification of the fibrosis area in the skeletal muscle of naïve (e) or infected mice submitted to treatment with saline (f), MSCs (g), or MSC_IGF-1 (h). Bars = 50 *μ*m. Quantification of inflammatory cells by morphometry (i) and evaluation of *Ptprc* expression by RT-qPCR (j). (k) Percentage of the fibrosis area quantified by analysis of whole sections stained with Sirius Red-stained skeletal muscle. Data represent the mean ± SEM of 5–7 mice per group. ^∗^*P* < 0.05, ^∗∗^*P* < 0.01, ^∗∗∗^*P* < 0.001, and ^#^*P* < 0.0001.

Muscle sections of naïve mice presented low interstitial cellularity (Figure 6(a)), while *T. cruzi*-infected mice were infiltrated predominantly by mononuclear cells between myofibers and surrounding blood vessels (Figures 6(b)–6(d)). Significant deposition of fibrosis was also observed in skeletal muscle sections of infected mice, compared to naïve controls (Figures 6(e)–6(h)). However, morphometric analysis did not show a reduction in the number of infiltrating inflammatory cells in mice treated with either MSCs or MSC_IGF-1, when compared to saline-treated mice. In fact, we found that mice treated with MSC_IGF-1 presented a significantly higher number of inflammatory cells (Figure 6(i)). This was corroborated by RT-qPCR analysis for the detection of PTPRC mRNA, demonstrating a significantly higher expression level for CD45, a panleukocyte marker (Figure 6(j)). The quantification of fibrosis by morphometric analysis in Sirius Red-stained sections showed that both treatments with MSCs and MSC_IGF-1 were able to reduce fibrosis in the skeletal muscle (Figure 6(k)).

**Figure 7:**
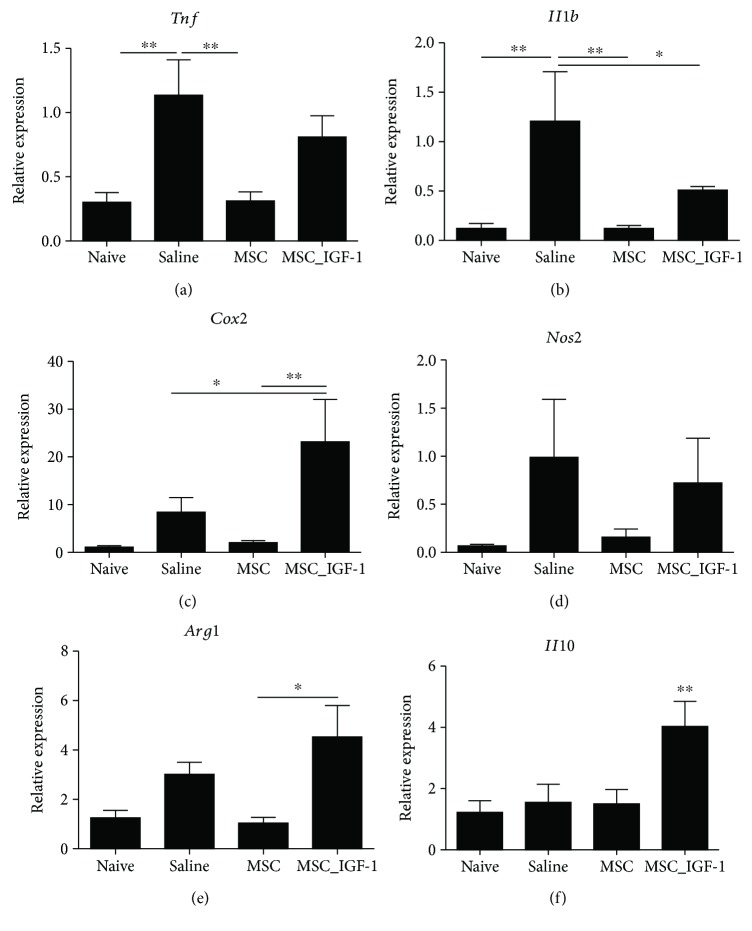
Gene expression analysis in the skeletal muscle. Skeletal muscle samples of uninfected or chagasic mice treated with MSCs, MSC_IGF-1, or saline were removed two months after therapy and analyzed by RT-qPCR for the expression of *Tnf* (a), *Ilb* (b), *Cox2* (c), *Nos2* (d), *Arg1* (e), and *Il10* (f). Data represent the mean ± SEM of 5–7 mice per group. ^∗^*P* < 0.05 and ^∗∗^*P* < 0.01.

Finally, gene expression analysis of the skeletal muscle tissue revealed that treatment with MSCs was able to significantly reduce the expression of proinflammatory cytokines TNF-*α* and IL-1*β* when compared to saline-treated mice. This was not observed for MSC_IGF-1-treated mice, which showed no significantly different expression levels, when compared to saline-treated mice (Figures 7(a) and 7(b)). In fact, MSC_IGF-1-treated skeletal muscle had increased expression of COX2 gene (Figure 7(c)). MSC_IGF-1 treatment did not significantly alter the expression of iNOS and arginase genes, markers of M1 and M2 macrophages, respectively (Figures 7(d) and 7(e)). Finally, a significantly higher expression of the IL-10 gene was seen in the MSC_IGF-1 group compared to the other groups (Figure 7(f)).

## 4. Discussion

In the present study, we evaluated whether IGF-1 overexpression can increase the immunomodulatory and/or regenerative actions of MSCs in the context of experimental Chagas disease cardiomyopathy. We found that transplantation of MSC_IGF-1 reduced cardiac inflammation and fibrosis, in a similar magnitude to the effect observed in MSC-treated mice. When skeletal muscle tissue was evaluated, however, a marked regenerative effect was observed in mice transplanted with MSC_IGF-1 cells.

IGF-1 has been associated with processes involved in both cardiac and skeletal muscle regeneration [7, 8, 20]. These studies, however, have focused on the role of IGF-1 in the regeneration after acute injuries to the skeletal muscle, while its role in a chronic setting, in the presence of persistent myositis, has not been addressed. We have previously shown that IGF-1 gene expression is increased in the heart and skeletal muscles in the acute phase of infection with *T. cruzi* in mice [21]. Here, we found that MSC_IGF-1 had a clear effect in the regeneration of the skeletal muscle from mice chronically infected with *T. cruzi*, in which the loss of skeletal myofibers could be the result of direct damage induced by *T. cruzi* infection associated with sarcopenia induced by chronic inflammation and increased local and circulating levels of TNF-*α* [22]. The fact that circulating TNF-*α* levels were equally modulated by both MSCs and MSC_IGF-1 suggests that the recovery of skeletal myofibers by IGF-1-overexpressing cells is not a consequence of immunomodulatory actions but possibly be the result of direct actions mediated by IGF-1 in the skeletal muscle.

The role of IGF-1 in the activation of satellite cell and muscle regeneration is well established [23]. It is also known that inflammation is crucial for muscle repair, and macrophages play an important role in this process by secreting IGF-1 to promote activation and proliferation of Pax7^+^ satellite cells [24]. Interestingly, recruitment of bone marrow cells to the skeletal muscle was also previously shown to contribute to myogenesis in a Pax7-/Myod-independent way [25]. Moreover, we have previously demonstrated the direct contribution of bone marrow-derived cells in the regeneration of myofibers during *T. cruzi* infection, in a bone marrow chimera experimental model [21].

The interaction between immune cells and muscle regeneration has been previously explored, and suppression of macrophage activity impairs muscle regeneration, leading to severe fibrosis [26]. Macrophage subtypes are also associated with different stages of the myogenic program, since proinflammatory (M1) macrophages predominate during the proliferative stage of muscle regeneration and anti-inflammatory (M2) macrophages are involved during the differentiation stage [27]. In the present study, therapy with MSC_IGF-1 was associated with increased numbers of mononuclear cells infiltrating the skeletal muscle, along with high expression of inflammatory mediators, such as TNF-*α* and IL-1*β*, along with the anti-inflammatory cytokine IL-10. These results could be explained by direct actions of IGF-1, but it is also possible that immune response directed towards the transgenes—GFP and hIGF-1—could have been elicited, and this was not investigated in the present study.

An interesting finding was the increased expression of COX2 in the skeletal muscle tissue of MSC_IGF-1-transplanted mice. IGF-1 signaling can induce COX2 expression in different cell types, including keratinocytes [28], mammary glands [29], and various tumor cells [30–32]. COX2 is an enzyme involved in arachidonic acid metabolism, responsible for the production of eicosanoids, including tromboxanes and prostaglandins, which are important inflammatory mediators. Importantly, prostaglandin E2 has been demonstrated to play a crucial role in processes of myoblast proliferation and regeneration, and functional recovery has been demonstrated [33, 34]. We found the expression of hIGF-1 in the muscle tissues of transplanted mice, indicating that this factor may be locally involved in the COX2 upregulation observed in our study and suggesting the participation of PGE2 in the muscle regeneration promoted by MSC_IGF-1 in chronic chagasic mice.

Several cell types have been investigated in therapies directed towards Chagas disease. Bone marrow mononuclear cells were initially tested in preclinical studies and were associated with a reduction in cardiac inflammation and fibrosis [35], mainly due to their immunomodulatory action [36], but failed to significantly improve the heart function in a randomized clinical trial [37]. MSCs have also been previously studied in experimental models of Chagas disease and were associated with significant improvements in inflammation and fibrosis [16, 18]. One significant limitation of our mouse model of Chagas disease is the lack of left ventricular dysfunction, which does not allow for the investigation of functional cardiac improvements that could be associated with the therapy. This limits our conclusions to aspects of immunomodulation, fibrosis, and regeneration, which were evaluated at the tissue level. Additional preclinical studies using a different model would be necessary to evaluate the potential of this gene and cell therapy in improving left ventricular function. To date, the results of clinical trials with MSCs in the treatment of heart failure of other etiologies have been modest, at best [38]. However, no clinical studies with MSCs in Chagas disease patients with cardiac dysfunction have been performed so far.

In conclusion, our results indicate that the overexpression of growth factors may be an interesting approach for improving the therapeutic potential of MSCs, since IGF-1 overexpression promoted increased proregenerative actions in association with maintained immunomodulatory and antifibrotic actions, when compared to regular MSCs, in the mouse model of chronic Chagas disease.

## Data Availability

The data used to support the findings of this study are included within the article.
